# A systematic review on deep learning‐based automated cancer diagnosis models

**DOI:** 10.1111/jcmm.18144

**Published:** 2024-03-01

**Authors:** Ritu Tandon, Shweta Agrawal, Narendra Pal Singh Rathore, Abhinava K. Mishra, Sanjiv Kumar Jain

**Affiliations:** ^1^ SAGE University Indore India; ^2^ Indore Institute of Science and Technology Indore India; ^3^ Acropolis Institute of Technology & Research Indore India; ^4^ Molecular, Cellular and Developmental Biology Department University of California Santa Barbara Santa Barbara California USA; ^5^ Electrical Engineering Department Medi‐Caps University Indore India

**Keywords:** cancer diagnosis, CNN, deep learning, machine learning, medical imaging, RNN

## Abstract

Deep learning is gaining importance due to its wide range of applications. Many researchers have utilized deep learning (DL) models for the automated diagnosis of cancer patients. This paper provides a systematic review of DL models for automated diagnosis of cancer patients. Initially, various DL models for cancer diagnosis are presented. Five major categories of cancers such as breast, lung, liver, brain and cervical cancer are considered. As these categories of cancers have a very high percentage of occurrences with high mortality rate. The comparative analysis of different types of DL models is drawn for the diagnosis of cancer at early stages by considering the latest research articles from 2016 to 2022. After comprehensive comparative analysis, it is found that most of the researchers achieved appreciable accuracy with implementation of the convolutional neural network model. These utilized the pretrained models for automated diagnosis of cancer patients. Various shortcomings with the existing DL‐based automated cancer diagnosis models are also been presented. Finally, future directions are discussed to facilitate further research for automated diagnosis of cancer patients.

## 1 INTRODUCTION

Cancer is considered as one of the most dangerous diseases in the world. Cancer is caused by the combination of genetic, environmental and lifestyle factors. In developing countries such as India, cancer is responsible for the maximum mortality rate with about 0.3% death per year.^1^ For the correct diagnosis and treatment planning early detection of cancer plays a very important role. It is a tedious task for the radiologists, oncologists and pathologists. Detection of cancer at the initial stages can improve the percentage rate of cured patients and hence the survival rate. Medical imaging techniques like magnetic resonance imaging (MRI), X‐ray and computed tomography (CT) are used most widely by medical practitioners for detecting cancer.^2^ Detecting cancer manually through biopsy images may be biased and may have varied opinions from doctors to doctors depending on their expertise and the parameters like exact and correct quantitative procedures to classify the images as normal or cancerous one. Automated system to identify cancer through microscopic images can play a significant role to reduce human errors, dependency and time and can provide better results.^3^

Use of Machine Learning (ML) algorithms for cancer detection with medical imaging and feature extraction is gaining high popularity. So, for detecting the different types of cancers like breast, lung, skin, liver, cervical and brain, various types of feature extraction techniques have been reviewed in medical imaging.^4^, ^5^ The previous studies show that the techniques used for feature descriptors from medical imaging expending the ML techniques have certain limitations, resulting in under performance of the software system.^6^ Deep Learning (DL) is a sub domain of ML with the extra characteristics of learning features directly through the available data set. Recent research shows that using the CNN model, DL techniques achieved improved accuracy for various types of cancer detection and diagnosis.^7^

In the real world, the most difficult task is to collect massive amounts of data in a variety of formats from a number of sources. When data are in unstructured format, traditional database management systems struggle to extract knowledge from it. It becomes necessary to manage both structured and unstructured data in order to create an effective system. Here, big data technology solves the problem by extracting knowledge from both structured and unstructured data.^8^

The present review work provides a comprehensive and systematic review of automated computational deep learning‐based models for cancer diagnosis. In addition, a thorough comparison of competitive automated computational models for cancer diagnosis is also considered. Discussion on different tools employed in developing automated computational models for cancer diagnosis is drawn. Further, identification and presentation of the shortcomings of competitive automated computational models for cancer diagnosis is included. Lastly, discussions on potential future research directions in the field of automated cancer diagnosis models is given.

*Research questions*:

The main purpose of this systematic review is to analyse reviews and surveys published between 2016 and 2022 in the area of deep learning in medical imaging. In doing so, we defined the following main research questions for our study:

1. Which deep learning‐based learning technique has consistently produced noteworthy prediction outcomes?
2. Which cancer, type of data and imaging techniques are used for cancer nodule detection.
3. Which AI‐based methods have consistently produced noteworthy prediction outcomes?
4. Which objective is mostly used to find the role of deep learning in cancer prediction?
5. Which data set and mode (online and offline) are mostly used in prediction of cancer?

*Main contribution*:

The main contributions of this study are as follows:

1. A systematic review of various automated computational deep learning‐based cancer diagnosis models is presented.
2. Comparison are drawn among competitive automated computational cancer diagnosis models.
3. Various tools that have been used to build automated computational models are also discussed.
4. Various shortcomings of the competitive automated computational cancer diagnosis models are presented.
5. Finally, future research directions in the field of automated cancer diagnosis models are discussed.

### 1.1 Image processing for cancer detection and diagnosis

**FIGURE 1:**
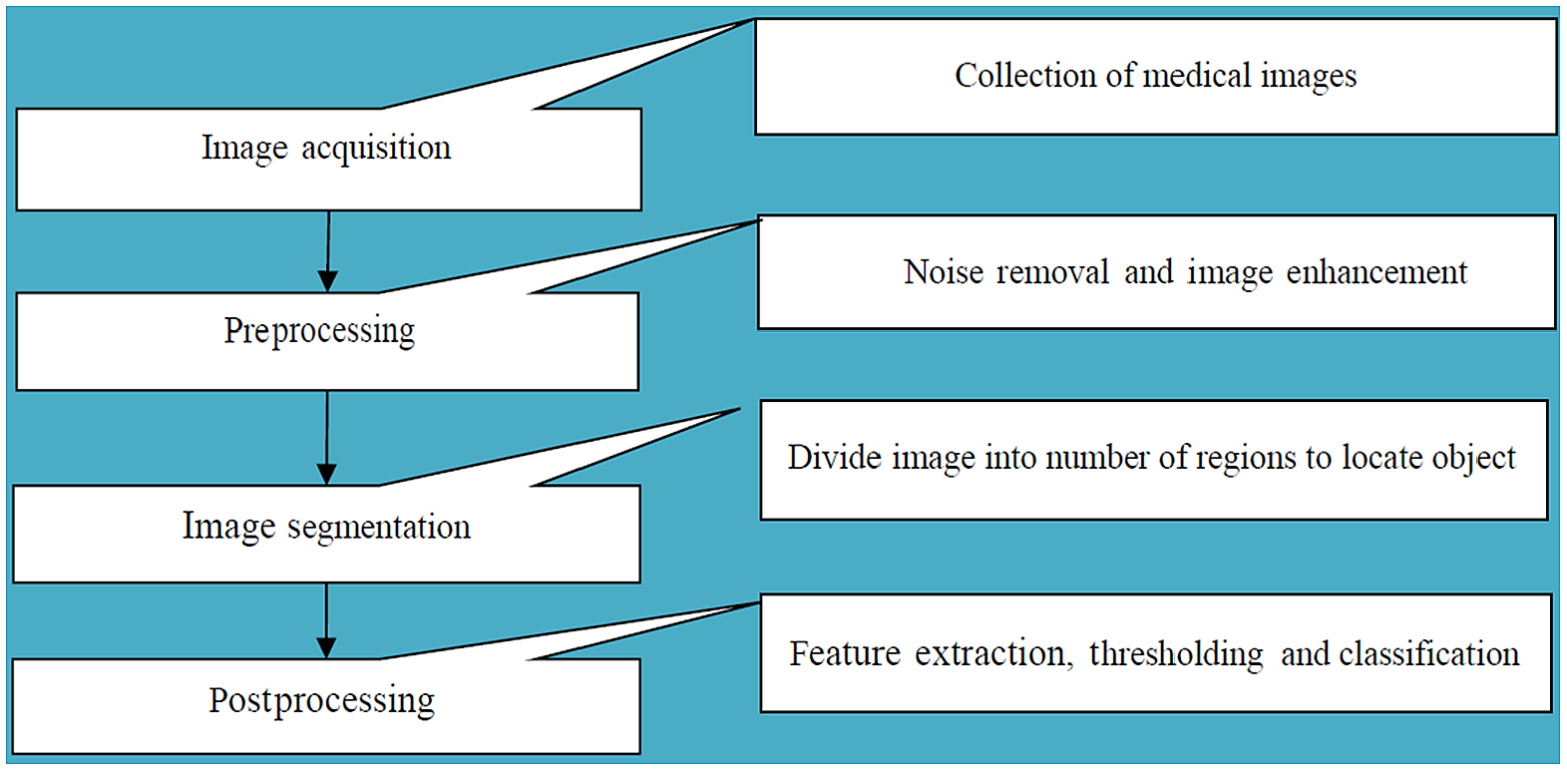
Schematics of steps in image processing for cancer detection.

Image processing techniques are being used widely in various domains of medicine. It includes disease detection, patient history analysis, drug discovery, conversation analysis, cancer detection and identifying cancer stages. From these stages noise removal, image enhancement and image segmentation from the medical images like CT scans and MRI are critical steps to decide the right treatment at the right time. Bhaskar Rao et al. used Berkeley wavelet transformation based segmentation method for reducing the image segmentation complexity process and improving the performance for brain tumour detection. Relevant features are extracted from each segmented region using the support vector machine classifier. Their experimental result showed an accuracy of 95.61%.^9^ Wason et al. presented the image processing technique for detecting lung cancer through CT images at an early stage. By using extracted features a classification model is applied to classify the tumour as benign or malignant.^10^ Figure 1 shows the various stages of image processing.

### 1.2 Artificial neural network (ANN)

Human brain has millions of neurons, which are the basic unit of the brain and the nervous system. These neurons receive sensory inputs via dendrites, process these inputs and output is produced through axons. ANN is a system inspired by the human brain that mimics human brain's behaviour in terms of learning new things that replicate the way humans learn. These neural networks learn from the examples and the experience and they do not require any set of instructions or the programming to perform any task. ANN consists of layers which are input, output and hidden layers. The number of hidden layers may vary depending upon architecture and application requirements. Mao et al. presented the evolution, working principle and properties of artificial neural networks (ANNs), as well as the current state of research into the use of ANNs in the detection and diagnosis of gastrointestinal and liver malignancies.^11^

### 1.3 DL in cancer detection and diagnosis

DL is a sub domain of ML and simulates the behaviour of human brain for learning patterns and data processing for taking decisions. DL is also termed as deep neural network or ANN with more hidden layers and complex structure.^11^ Many studies have been employed in detection of cancer using different approaches and models of image processing and DL. Due to the availability of huge medical data, systems with high computational power and GPUs the developments of learning algorithms utilizing DL is gaining importance for disease diagnosis in the healthcare sector and particularly in oncology care.^12^ The features of DL like image segmentation, image labelling, pattern recognition and object detection makes it suitable for applications in radiology.

DL techniques are widely used for tumour, lesion detection, classification and image segmentation. Stacked Ensemble Model (stacking of bagged and boosted learners) for disease diagnosis automation is proposed by authors.^13^ M. Coccio et al. presented a review on use of DL algorithms in health informatics and analysed various benefits and potential consequences of these algorithms.^14^ Gupta et al. presented and compared various deep learning models for prediction and diagnosis of colon cancer. Deep auto encoders had the highest performance with 97% accuracy and 95% area under curve‐receiver operating characteristic (AUC‐ROC).^15^ Kanavati et al. presented the classification of WSIs of LBC specimens into malignant and non‐neoplastic using a deep learning model. For the same a data set of 1605 cervical WSIs are used.^16^

### 1.4 Convolutional neural network (CNN)

CNN is a DL algorithm that is popular in computer vision applications and applicable to varied domains of healthcare. CNN is gaining popularity in image processing because of its dimensionality reduction characteristics. CNN is composed of convolution layers, pooling layers and fully connected layers which learn features in hierarchical manner through back propagation.^14^ CNN in radiology studies show that for feature extraction, hand crafted techniques such as texture analysis and the conventional ML classifier support vector machine are used. Whereas CNN does not require hand crafted feature extraction from the images and the manual segmentation of images for finding of any tumour.

**FIGURE 2:**
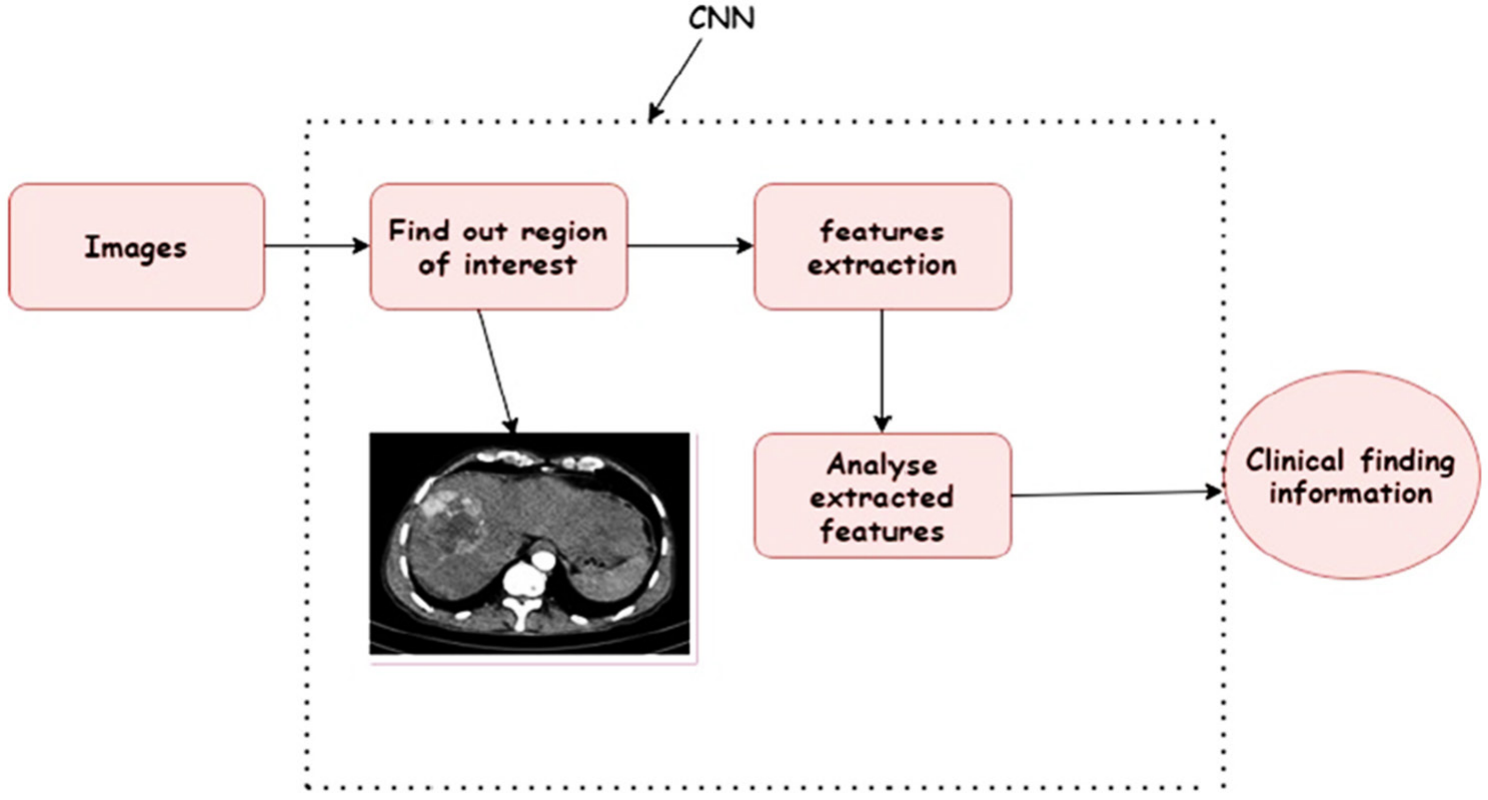
Schematics of feature extraction and diagnosis with large data set ROI finding.

Radiologists have a large amount of data and extraction for region of interest and features of the tumour as quantitative parameters is shown in Figure 2. As the features are expressed by continuous variables, so it has the potential to capture more features as compared to the visual assessment. CNNs are suitable for these cases of medical imaging for radiologists because of the huge amount of data availability and large number of features.^17^ Use of a pruned CNN model is proposed for the diagnosis of Lumbar Spondylitis in.^18^ The other advantage of CNN is automatic extraction of features through input images.

In the following section, discussion regarding the methodology used for cancer identification is presented. Section 3 presents a comprehensive review of breast cancer, lung cancer, brain cancer and liver cancers. Section 4 focuses on the discussion for prediction outcomes based on deep learning techniques. Finally, Sections 5 and 6 addresses the challenges encountered throughout the research with key findings summarization and proposed future research directions.

## 2 RELATED WORK

### 2.1 Process flow for the DL based data analysis

**FIGURE 3:**
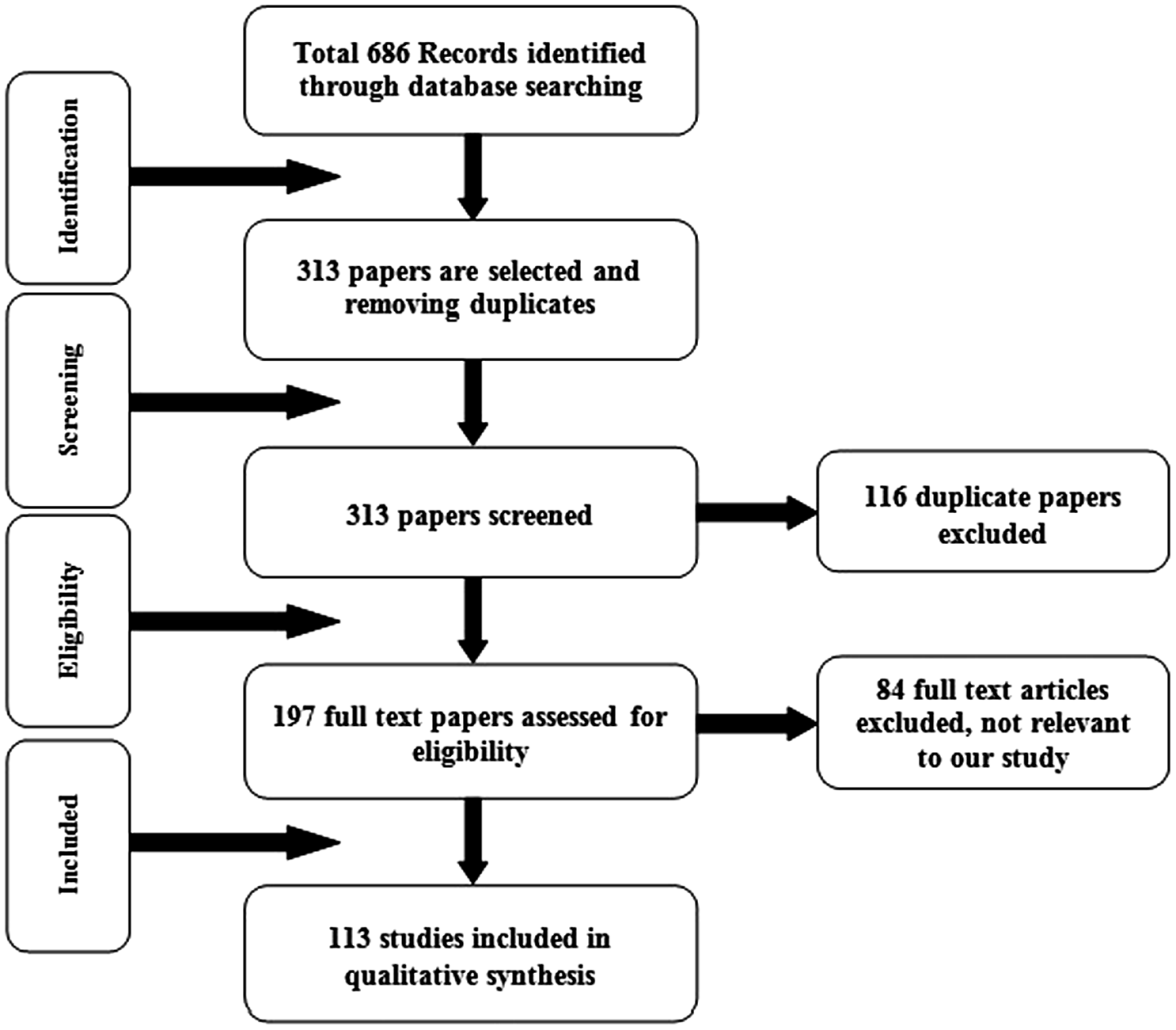
Process flow of review analysis (PRISMA) for included articles.

This systematic review presents the use of DL techniques for the five major types of cancers: breast cancer, lung cancer, brain cancer, cervical cancer and liver cancer. Figure 3 shows the process flow of review analysis (PRISMA) for included articles.

The methodology of review consists following steps:

#### 2.1.1 Step 1. Data collection

**FIGURE 4:**
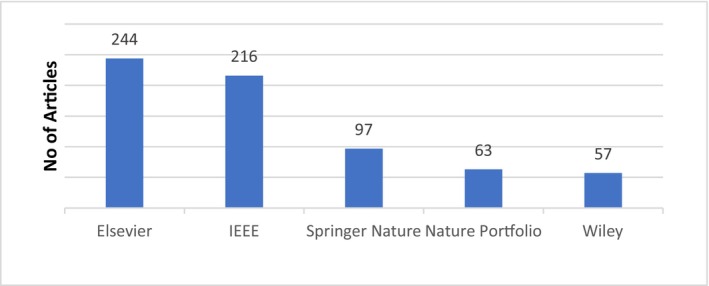
Summary of selected articles from five databases.

**FIGURE 5:**
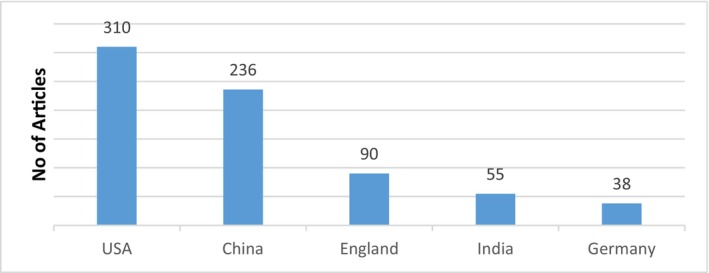
Top Five countries with publications related to automated cancer diagnosis.

Five databases, that is, Elsevier, Scopus, Springer Nature, Wiley and Institute of Electrical and Electronic Engineers (IEEE) Digital Library were searched from 2016 to 2022. Figures 4 and 5 show the databases and articles considering cancer detection using DL within this period from various databases and countries.

1. *Search Terms*: For survey defined search terms were as follows: ‘computer vision’ or ‘machine vision’ or ‘artificial intelligence’ or ‘machine learning’ or ‘DL’ AND breast cancer or lung cancer or cervical cancer or brain tumour or liver cancer.
2. *Inclusion criteria*: To search the relevant articles, first the titles and abstracts were searched. Once meeting the search terms criteria and then duplicate papers were removed.
3. *Exclusion criteria*: Articles that did not present techniques of cancer detection and diagnosis through DL were excluded from the study.

#### 2.1.2 Step 2. Data analysis

In this review work a total of 686 papers were selected from Web of Science (WoS) collection. Data analysis is performed with following considerations,

1. *Year of Publication*: As DL for cancer detection has mostly attracted researchers from the past decades, publications from 2016 to 2022 for cancer detection using deep learning is considered.
2. *Purpose of the study*: Different types of task performed by DL for selecting the types of cancers such as lesion detection, classification, segmentation etc. is considered.
3. *Image Acquisition Technique*: The types of medical imaging techniques such as MRI, CT scan and chest X‐ray are used in different studies.
4. *DL Architecture*: Considered DL architectures such as CNN, Deep belief network, Fast CNN and Recurrent Neural Network are the important architectures in the field of medical for various types of applications.
5. *Image Data set*: Various types of Image data sets for cancer detection and diagnosis is presented and summarized.

#### 2.1.3 Step 3. Application domain

**FIGURE 6:**
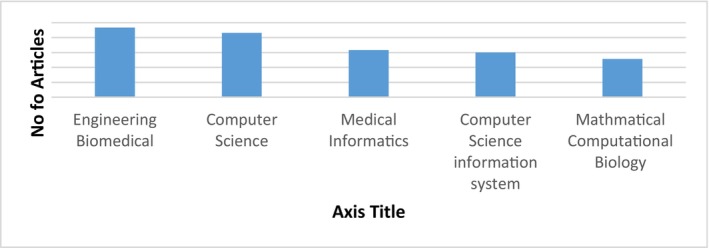
Application domains of deep learning models for cancer detection.

1. Studies from different domains that have been implemented using various DL models for cancer diagnostics are summarized in Figure 6.

## 3 USE OF DL TECHNIQUES FOR THE CANCERS DIAGNOSIS

### 3.1 Breast cancer

**FIGURE 7:**
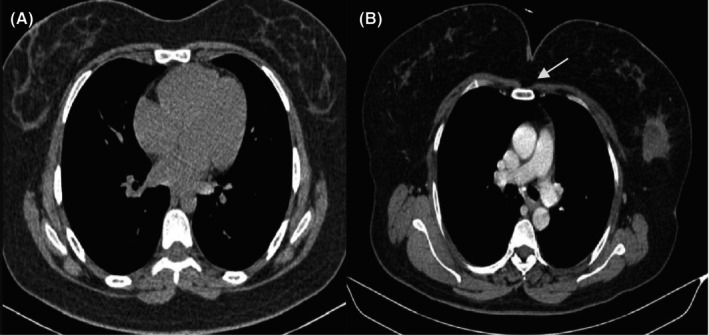
(A). Normal Breast (B). Breast malignant mass. Arrow indicate the malignant mass.

In recent years, various DL applications for breast cancer detection and diagnosis have been used. Albayrak et al. implemented feature extraction algorithm using DL models with accuracy of 96% to detect mitosis in histopathological images of breasts.^19^ An image of MRI scan of normal and cancerous breast is shown in Figure 7.

Spanhol et al. presented CNN model for classifying breast histopathological images through Break‐ His data set. Authors utilized AlexNet architecture for design and implementation of CNN for differentiating benign tumours and malignant tumours.^20^ A deep network for detection of mitosis for cancer detection using breast histology images by training a fully convolutional network (FCN) model to take out mitosis from the whole histology images is proposed by Chen et al.^21^ Albarqouni et al. presented a deep convolutional network for nonexpert crowd explanation in biomedical context. The authors developed multiscale CNN architecture to present crowd annotations.^22^ A method by using unlabeled data for learning the features hierarchy and learned features for performing two different tasks that are breast density segmentation and scoring of mammographic texture is presented by Kallenberg et al.^23^

Selvathi et al. used mammogram images using an unsupervised method of deep learning for breast cancer detection. Stacked auto encoder and Softmax classifier were used to form deep networks.^24^ Habibzadeh et al. proposed the Res‐Net architecture of CNN to detect breast cancer with specific settings as compared to the conventional approach. The accuracy of the proposed Res‐Net architecture is 99.8% and 99.7% for benign and malignant breast cancer.^25^ A classification model for categorizing whole side images (WSI) in breast biopsies in different diagnostic classes is proposed by Gecer et al.^26^ Authors have used the FCNN classifier for labelling of the WSI. Xie et al. have adapted Inception_V3 and Inception_ResNet_V2 architectures to classify breast cancer histopathological image for the binary‐ and multiclass issues by utilizing transfer learning techniques. Results show that the classification of breast cancer using Inception_V3 and Inception_ResNet_V2 is superior to the existing methods.^27^ Le et al. formed a map of WSI that is providing patterns and spatial distribution of lymphocytic infiltrates and improving quantification of TILs. Authors have used three CNN architectures that are Resnet‐34, Inception v4 and VGG16 for TILs analysis and segmentation.^28^ Kaur et al. used Mini‐MIAS data set consisting of 322 images and using K‐mean clustering. Results showed appreciable accuracy with *K*‐Mean clustering and MSVM as compared to decision trees model.^29^

Toğaçar et al. reconstructed the data set for the processing of the auto encoder model and showed 95.89% classification success rate.^30^ A KNN algorithm along with an auto encoder is used for the detection of breast cancer disease with 91.24% accuracy in.^31^ Gandomkar et al. proposed a Deep Residual Network model consisting of 152 layers to classify the images as benign or malignant and achieved classification accuracy of 98.52%, 97.90% and 98.33%, respectively.^32^ A CNN model for early detection of cancer using thermal breast images optimized by bays algorithm and achieved accuracy of 98.95%.^33^, ^34^

Voting classifier (VC), logistic regression (LR), decision tree (DT), random forest (RF), support vector machine (SVM), and a proprietary convolutional neural network were used to construct prediction models (CNN) by Mohammad Monirujjaman et al. and realized an average accuracy of 99%^35^, ^36^ Table.1.

**TABLE 1:** Summary of the studies for breast cancer detection and diagnosis using DL models.

Reference	Year	Objective	Image type	DL architecture and methods	Data set	Accuracy
Albayrak et al.^19^	2016	Cancer detection	Histopathology	CNN	MITOS ATYPIA‐14	96%
Snaphol et al.^20^	2016	Classification	Histopathology	CNN Alexnet transfer learning	BreaKHis	85% to 90%
Albarqouni et al.^22^	2016	Mitosis detection	Histopathology	CNN AggNet	MICCAI‐AMIDA‐13	Not defined
Kallenberg et al.^23^	2016	Image classification	Mammographic	CSAE (convolutional sparse autoencoder)	Unpublished clinical data set	Not defined
Altameem et al.^24^	2022	Breast cancer detection	Mammographic	Fuzzy ensemble	Mini‐MIAS +3	99.32%
Motlagh et al.^25^	2018	Classification	Histopathology	CNN ResNet transfer learning	Tissue microarray and BreakHis	96.4%
Gecer et al.^26^	2018	Classification	Whole‐slide breast histopathology	CNN and FCN transfer learning	NIH Sponsored project	Not defined
Xie et al.^27^	2019	Clustering	Histopathology	CNN Inception_V3 and Inception_ResNet_V2 transfer learning	BreaKHis	76.4% and 59.3%
Le et al.^28^	2019	TIL analysis and Classification (Tumour Infiltrating Lymphocytes)	Whole‐slide tissue images	CNN Resnet‐34, VGG16 and Inception v4 transfer learning	SEER	89%
Kaur et al.^29^	2019	Classification	Mammographic	CNN and MSVM	Mini‐MIAS	95%
Toğaçar et al.^30^	2019	Classification	Histopathology	Autoencoder	IDC data set	98.59%
Adem et al.^31^	2019	Classification	Tabled data	Autoencoder	Kent Ridge‐2 database	91.24%
Gandomkar et al.^32^	2018	Classification	Histopathology	Deep ResNet transfer learning	BreaKHis	95.40%, 94.90% and 95.70%
Ekici et al.^33^	2019	Classification	Thermal breast images	CNN	unpublished	98.95%
Naderan et al.^34^	2020	Breast cancer detection	Histopathology	Autoencoder	BreaKHis	84.72%
Mohammad Monirujjaman et al.^35^	2022	Breast cancer detection	Tabled data	LR, RF, SVM, VC, DT and a custom CNN model.	Kaggle	99%
Xiaomei Wang et al.^36^	2022	Breast cancer detection	BC‐IDC data set	CNN and GRU	Kaggle	86.21%

### 3.2 Lung cancer

**FIGURE 8:**
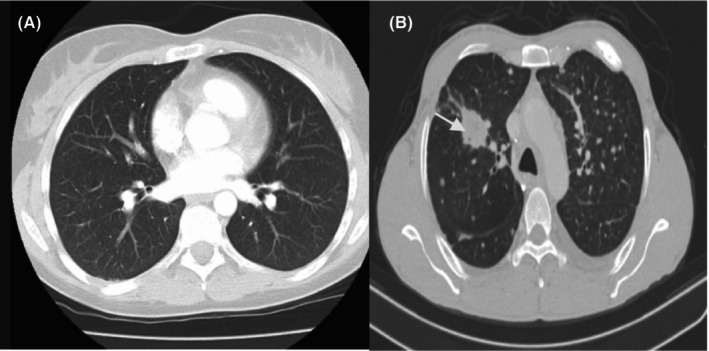
(A) Normal lung. (B) Carcinoma lung right side. Arrow indicates the malignant mass.

Early detection of lung cancer may be done through the various imaging techniques like chest X‐ray, CT scan and MRI. For the detection of respiratory disease X‐ray imaging is the commonly used technique.^37^, ^38^ A representative image is provided in Figure 8.

In the CT scans multiple doses of radiation can increase the chances of cancer. So, Wei presented an adaptive partial differential equation model to reconstruct the images using fewer CT images to decrease the radiation dose.^39^ Capizzi et al. used the fuzzy system with the neural network for the evaluation of lung screening model.^40^ Figure 8A,B shows the pictures of normal lung and the carcinoma lung, respectively. Zhu et al. developed a deep CNN model named Deep ConvSurv for survival analysis using pathological images. Using this Deep ConvSurv model, end‐to‐end survival can be predicted using pathological images.^41^

Hussein et al. developed a multiview CNN named Tumour NetData augmentation for determining nodule of malignant tumours.^42^ Shen et al. introduced a DL model of multicolumn convolutional neural network (MC‐CNN) to deal with the critical issue of lung lump malignancy classification. The encouraging results on lump malignant cells classification reflect the efficiency of MC‐CNN.^43^, ^44^ Tajbakhsh et al. proposed comparison between two classes of continuous ML massive training artificial neural networks (MTANNs) and CNNs. The results show that the performance of MTANNs is better than CNN with limited training data.^45^

Jung et al. presented a three‐dimensional deep convolutional neural network (3D DCNN) having shortcut connections and three dimensional Deep CNN for lung lump classification. The results show superior performance metrics using three dimensional deep convolutional networks.^46^ Woźniak et al. suggested a novel method for classifying lung carcinomas. For each pixel of an original picture, the local variance that gets the result image (‘variance image’) of the same image size begins with the locating and extraction of the lung nodule by calculating.^47^

Mao et al. proposed an unsupervised deep auto encoder for the classification of lung cancer.^48^ To ensure a truthful diagnosis of lung nodules, the proposed framework incorporates the following two categories of features, (i) appearance features modelled with a higher‐order Markov Gibbs random field (MGRF) model capable of describing spatial in homogeneities within the lung nodule and (ii) geometric features describing the shape geometry of the lung nodules.^49^ Vaishnavi et al. showed that in CT images, tumour segmentation and classification are challenging task. So, the authors proposed a method to automatically detect and classification of lung tumours tissues.^50^ Xiea et al. proposed an automatic pulmonary lump detection using two dimensional CNN for assisting the CT process using LUNA16 and achieved accuracy of 86.4%.^51^

Use of CT images for training double convolutional deep neural network (DCDNN) and a regular convolutional deep neural network (CDNN) is proposed by Jaki movski et al.^52^ The ADL model has multiple strategies for diagnosing malignant nodules efficiently utilized by Nasrullah et al. In the same, two deep three‐dimensional (3D) customized mixed link network (CMixNet) models have been used to detect and classify lung nodules.^53^ Silva et al investigated a transfer learning method based on unsupervised learning achieved by training a convolutional auto encoder (CAE) with images from the same domain.^54^ Ghosal et al proposed the use of a generative adversarial network (GAN) as a data augmentation strategy for increasing the amount of training data available to CAD systems.^55^

Amjad Rehman, et al. employed support vector machine and K‐ nearest neighbours for the classification of features from CT chest images.^56^ Lu, Xinrong et al. proposed an optimal methodology for detection of lung carcinoma at early stage. For efficient network accuracy and optimal arrangement Marine predators algorithm is utilized in.^57^ O. Obulesu et al. proposed a Wilcoxon Signed Generative Deep Learning (WS‐GDL) method for detection of lung cancer.^58^ Chan Zhang et al. used masked R‐CNN for image segmentation and processing of lung nodules.^59^ Table 2 shows the summary of the methods used for detection of lung cancer.

**TABLE 2:** Summary of the research papers for lung cancer detection and diagnosis using DL models.

Reference	Year	Objective	Image type	Methods	Data set	Accuracy
Zhu et al.^41^	2016	Survival analysis	Histopathology	CNN	NLST	12% improvement
Hussein et al.^43^	2017	Lump characterization	Volumetric CT	CNN	LIDC‐IDRI	92.31%
Shen et al.^42^	2017	Lung nodule suspiciousness classification	Volumetri CT	CNN	LIDC‐IDRI	87.14%
Wang et al.^45^	2016	Lung tumour classification	CT slices	Transfer learning	JSRT	Not defined
Tajbakhshet al.^43^	2017	Lump detection and classification	CT slices	MTANN and CNN	Unpublished Data set	95%
Jung et al.^46^	2018	Lung tumour classification	CT slices	3DCNN transfer learning	LUNA‐16	Not defined
Wozniak et al.^47^	2018	Small lung nodules detection	X‐ray	Probabilistic neural network	Not defined	92%
Mao et al.^48^	2018	Lung tumour classification	CT scans	Autoencoder	ELCAP	Not defined
Shaffie et al.^49^	2018	Lung tumour detection	CT scans	Autoencoder	LIDC‐IDRI	92.20%
Vaishnavi et al.^50^	2019	Lung tumour detection	CT images	Probabilistic neural network	Unpublished data set	Not defined
Xiea et al.^51^	2019	Automated nodule detection	CT images	2D CNN transfer learning	LUNA‐16	86.42
Jakimovski et al.^52^	2019	Lung nodule diagnosis	CT images	CDNN	LONI data set	99.62%
Nasrullah et al.^53^	2019	Automatic lung nodule detection	Chest X‐ray and CT images	Recurrent CNN (RCNN)	LUNA16 and LIDC‐IDRI	94.17%
Silva et al.^54^	2020	Lung nodule detection	CT images	Autoencoder	LIDC‐IDRI	0.936 AUC
Ghosal et al.^55^	2020	Lung nodule detection and classification	CT images	Convolutional autoencoder	LIDC‐IDRI	95.3%
Rehman, Amjad, et al^56^	2021	Detection and classification of lung cancer	CT Images	SVM and KNN	VIA ELCAP	93% and 91%
Lu, Xinrong et.al^57^	2021	Lung cancer detection	CT Images	Deep learning	RIDER	93.4%
Obulesu, O., et al^58^	2021	Lung cancer diagnosis	CT Images	WS‐generative deep learning	Unpublished	86%
Zhang, Chan, et al^59^	2021	Lung nodule detection	CT images	CNN	Private hospital	93.75%

### 3.3 Brain tumour

**FIGURE 9:**
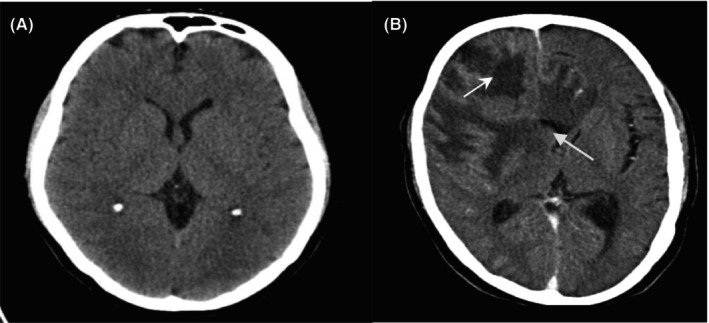
(A) Normal Brain. (B) Brain tumour. (arrow indicate high grade glioma).

Many DL methods have been proposed for diagnosing and detecting tumours in the brain through CT and MRI. Pereira et al.^60^ discussed limitations requiring large amount of time for manual segmentation of MRI of glioma. Figure 9A,B show the images of normal brain and brain tumour (high grade glioma), respectively.

A deep neural network to detect brain tumours for customization of both low and high grade (glioma) pictures in MRI images is proposed by Havaei et al.^61^ Gao et al. introduced the early diagnosis of Alzheimer's disease. In the same, authors classified CT images into three cluster groups that are Alzheimer's disease (AD), Tumour and normal ageing and implemented advanced CNN architectures.^62^ Ehab et al. selected 44 Glioma images of patients in the two different categories in image archive data set.^63^ Ahmed et al. presented the study and implementation of DL model to predict rate of survival of brain tumour patients. Authors have tested their results on the very small data set due to unavailability of larger data set. Results show an accuracy of 81.8%.^64^, ^65^ A system using CNN is proposed for early detection of Glioma with approximate survival rate of 11–15 months and achieved 80% accuracy.^66^ Wang et al. proposed a cascade of CNNs in the MRI section of brain tumours, and introduced a 2.5D network that offers micro consumption, model complexity and reception fields MRIs.^67^ Amin et al. utilized a high pass filter image to emphasize in homogeneities of the MR slices' field effect, which was then fused with the input slices.^68^

Ali et al. proposed an effective approach to use residual networks for classifying brain tumour types. Accuracy of 99% is achieved by authors in comparison with earlier work on the same data set.^69^ Badža et al. presented a novel convolutional neural auto‐encoder for semantic segmentation of brain tumours.^70^ K. Aswani et al. proposed an unsupervised dual auto encoder with latent space optimization. The model is trained using only normal MRI images, which eliminates the need for a massive tumour database.^71^ Gupta et al. proposed convolutional neural networks (ConvNet) ensemble for segmenting glioma from magnetic resonance images.^72^ Ayesha Younis et al. proposed a faster convolutional feature maps using CNN using the VGG‐16 architecture as a primary network. These maps were subsequently categorized to produce suggestions for the tumour region.^73^ Table 3 shows the summary of the DL methods for detection of Brain tumours.

**TABLE 3:** Summary of the research papers for brain tumour detection and diagnosis.

Reference	Year	Objective	Type	Methods	Database	Accuracy
Pereira et al.^60^	2016	Segmentation of brain tumour	MRI	CNN	BRATS 2013 and 2015	Not defined
Havaei et al.^61^	2017	Segmentation of brain tumour	MRI	Deep CNN	BRATS 2013	30 times faster result
Gao et al.^62^	2017	Classification of brain tumour	CT Images	2D and 3D CNN	285 data set from navy hospital china	87.62%
Ehab et al.^63^	2017	Automatic segmentation of tumour	MRI	CNN	Cancer imaging archive	Dice coefficient above 0.8
Ahmed et al.^64^	2017	Classification of brain tumour	MRI	CNN	Unpublished	81%
Tandel et al.^66^	2019	Classification of brain tumour	MRI	CNN and ANN	BRATS 2018	80.03%
Wang et al.^67^	2019	Brain tumour segmentation	multimodal MRI	Cascaded transfer learning	BraTS 2017 and BraTS 2018	Not defined
Amin et al.v^68^	2019	Brain tumour detection	MRI	Stacked autoencoder	BRATS 2018	95%
Ali et al.^69^	2020	Brain tumour classification	MRI	CNN(ResNet) transfer learning	Nanfang Hospital	99%
Badža et al.^70^	2021	Brain tumour segmentation	MRI	Convolutional autoencoder	Nanfang Hospital, Guangzhou	99.23%
Aswani et al.^71^	2021	Brain tumour segmentation	MRI	Dual encoder	BraTS 2015	Not defined
Ayesha Younis et al.^73^	2022	Brain tumour diagnosis	MRI	CNN and VGG16	Private data set	CNN:96% and VGG: 98.5%

### 3.4 Cervical cancer

**FIGURE 10:**
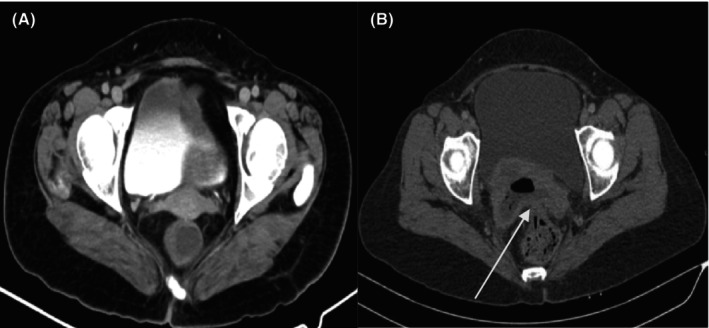
(A) Normal cervix. (B) Cervical carcinoma. (arrow indicating malignant mass).

A number of DL methods have also been proposed for the diagnosis and detection of cervical cancer. Figure 10A,B shows the images of normal cervix and cervical carcinoma, respectively. Song et al. proposed a segmentation method that could be used to detect and diagnose cervical cancer automatically by using super pixel and CNN method.^74^ Xu et al. proposed a technique for improving accuracy of precancerous cells that is cervical dysplasia. The authors have designed a DL framework using multimodal information.^75^

Devi et al. presented the cervical cancer detection and working using ANN architecture to classify normal or abnormal cervical cells.^76^ A convolutional‐based cervical cancer model for intelligent and efficient classification implemented by Wu et al.^77^ Alyafeai et al. proposed an automated pipeline to detect and classify cervical cancer through cervigram images.^78^ Their pipeline architecture is very much appropriate for mobile phone deployment with the higher speed and accuracy of 82%.

Pathania et al. developed a DNA‐focused digital micro holography method for HPV screening, with automated methods using deep‐learning algorithms. The result of this paper shows the excellent sensitivity and specificity (100% concordance) in detecting HPV 16 and 18 DNA from cell lines.^79^

Sompawong et al. presented the method for detecting cervical cancer employing Pap smear histology slides using the Mask Regional Convolution Neural Network (Mask R‐CNN). This is the first attempt to use the R‐CNN mask to identify and analyse the cervical cell nucleus, screen for normal and aberrant nuclear characteristics.^80^

Khamparia et al. for training and testing, they used the Herlev data set, which contains 917 cervical pap smear cells with 26 attributes and two target variables.^81^ Chen et al. developed an artificial intelligence (AI) system called CytoBrain to screen abnormal cervical cells automatically in order to aid in the subjects' subsequent clinical diagnosis.^82^ Chandran et al. developed a model CYENET to automatically classify cervical cancers based on colposcopy images with an accuracy of 73.3%.^83^ Ensemble approach of machine learning is used for the diagnosis of cervical cancer automatically.^84^ Using Boruta analysis and the SVM method, a model for effective feature selection and prediction for data sets related to cervical cancer was presented to address this issue.^85^ Table 4 shows the summary of the work done for detection of cervical cancer except review and survey articles.

**TABLE 4:** Summary of the research papers for cervical cancer detection and diagnosis using DL models.

Reference	Year	Objective	Image type	DL architecture and methods	Data set	Accuracy
Song et al.^74^	2016	Segmentation of cervical cell cytoplasm	Histopathology	CNN	Unpublished	91.83%
Xu et al.^75^	2016	Cervical abnormality diagnosis	Digital cervicography	CNN	Unpublished	88.91%
Wu1 et al.^77^	2019	Cervical cell classification	Cytological images	CNN	Unpublished	89.28%
Alyafeai et al.^78^	2019	Cervix detection and cervical cancer classification	Digital cervicography	CNN	Intel and mobile ODT data set	82%
Pathania et al.^79^	2019	Cervical cancer screening	Histopathology	CNN	Unpublished	Not defined
Sompawong et al.^80^	2019	Cervical cancer screening	Pap smear histological slides	Mask R‐CNN transfer learning	Thammasat University (TU) Hospital	89.8%
Khamparia et al.^81^	2020	Cervical cancer classification	Pap smear histological slides	Autoencoder	Herlev data set	99.2%
Chen et al.^82^	2021	Automated cervical cell screening	Cervical cell images	Autoencoder	Unpublished	98.02%
Chandran et al.^83^	2021	Cervical cancer detection	Colposcopic images	Autoencoder	Intel ODT data set	92.3%
Umesh Kumar Lilhore et al.^85^	2022	Cervical cancer detection	Colposcopic images	SVM	Tabular data	95%

### 3.5 Liver cancer

**FIGURE 11:**
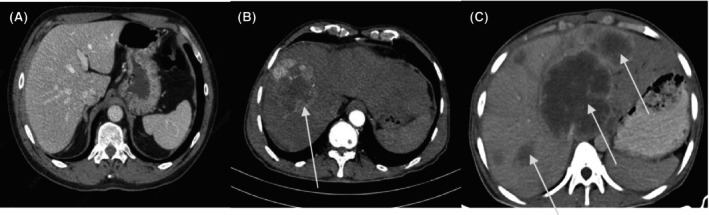
(A) Normal liver on CT scan. (B) Hepatocellular carcinoma. (C) Multiple liver lesions—Metastasis (arrows indicating the malignant lesion).

CT and other scans based on DL methods have been proposed for diagnosis and detection of liver cancer. Figure 11A shows the normal liver CT scan, Figure 11B shows the Hepato‐cellular carcinoma and Figure 11C shows the scan of multiple liver lesions—Metastasis.

Christ et al. presented a technique for liver and lesion segmentation automatically through CT images of abdomen by using the fully convolutional network model of DL.^86^ Cohen et al. proposed a method for detecting small lesions in CT images for liver cancer. Authors used a combined local and global approach in the system that can enhance the detection capabilities.^87^ Das et al. proposed a technique called watershed Gaussian based DL (WGDL) technique to find the cancer lesion in CT images of the liver. Authors have shown an achievement of total classification accuracy around 99.38%.^88^ Peng et al. proposed a DL model, that is trained and validated the preoperative prediction for the response of patients.^89^ A new three‐dimensional (3‐D) convolution network (CNN) is proposed by Trivizakis et al.^90^ Evaluation of the influence of images acquisition on transfer learning (TL) diagnosis by adopting pretrained neural convolution networks (CNCs) and three phased computed dynamic contrast tomography (DCE‐CT) is used for primary hepatic malignancies in.^91^, ^92^

Budak et al. presented two convolutional deep encoder‐decoder neural networks (EDCNNs). The networks are constructed and trained to cascade segments of the liver and lesions in CT images with limited image quantity.^93^ Lee et al. investigated liver imaging characteristics of stage I–III colorectal cancer (CRC) from 2019, using CNN for the oncology purposed.^94^ Dong et al. proposed HFCNN for segmenting the liver tumour.^95^ Almotairi et al. presented a DL model for classifying images and fitting liver segmentation. SegNet deep convolutional encoder–decoder architecture and accuracy up to 99.9% in the training phase is shown in.^96^ Hassan et al. presented a DL based feature representation technique with a stacked sparse auto‐encoder. A softmax layer classifier is used to classify the different important liver diseases and achieved classification accuracy of 97.2%.^97^

Roy et al. presented Histo‐CAE, a multiresolution deep learning model for segmentation of viable tumours in whole‐slide liver histopathology images.^98^ Also, some of the authors have shown efficient results for the cancer detections.^99^, ^100^, ^101^ Table 5 shows the summary of the work done for detection of Liver cancer except review and survey articles.

**TABLE 5:** Summary of the research papers for liver cancer detection and diagnosis using DL models.

Reference	Year	Objective	Image type	DL architecture and methods	Data set	Accuracy
Christ et al.^86^	2016	Liver and lesion segmentation	CT abdomen images	FCNN	3DIRCADb data set	93.1%
Cohen et al.^87^	2018	Liver metastases detection	CT images	FCNN transfer learning	Unpublished data set	94.7%
Das et al.^88^	2019	Automatic detection of liver cancer	CT images	DNN	Unpublished data set	99.38%
Peng et al.^89^	2019	DL model for preoperative prediction	CT images	Residual CNN	Unpublished data set	84.3%
Trivizakiset al.^90^	2019	Tissue classification in liver tumour	MRI	3D CNN	Collected from private hospital	83%
Vanmoreet al.^91^	2019	Estimation of level of liver damage	CT images	DCNN transfer learning	Not published	98.5%
Budak et al.^92^	2019	Liver tumour segmentation	CT images	Deep encoder‐decoder	3DIRCADb	Not defined
Dong et al.^93^	2020	Liver cancer detection	CT images	FCNN	Not published	97.22%
Almotairi et al.^96^	2020	Liver tumour segmentation	CT liver scans	DNN	3D‐IRCADb‐01 d	99.9%
Hassan et al.^97^	2020	Diagnosis of focal liver disease	Ultrasound images	FUZZY K MEAN CLUSTERING	Egyptian Liver Research Institute	97.2%
Roy et al.^98^	2021	Liver tumour segmentation	Whole side image	CNN auto‐encoder	PAIP challenge 2019	95%

## 4 DISCUSSION

In this review recent studies related to cancer detection using deep learning methods are presented. This article summarizes studies on cancer diagnostic and prediction using deep learning‐based approaches.

### 4.1 Consistent prediction outcomes based on deep learning techniques

**FIGURE 12:**
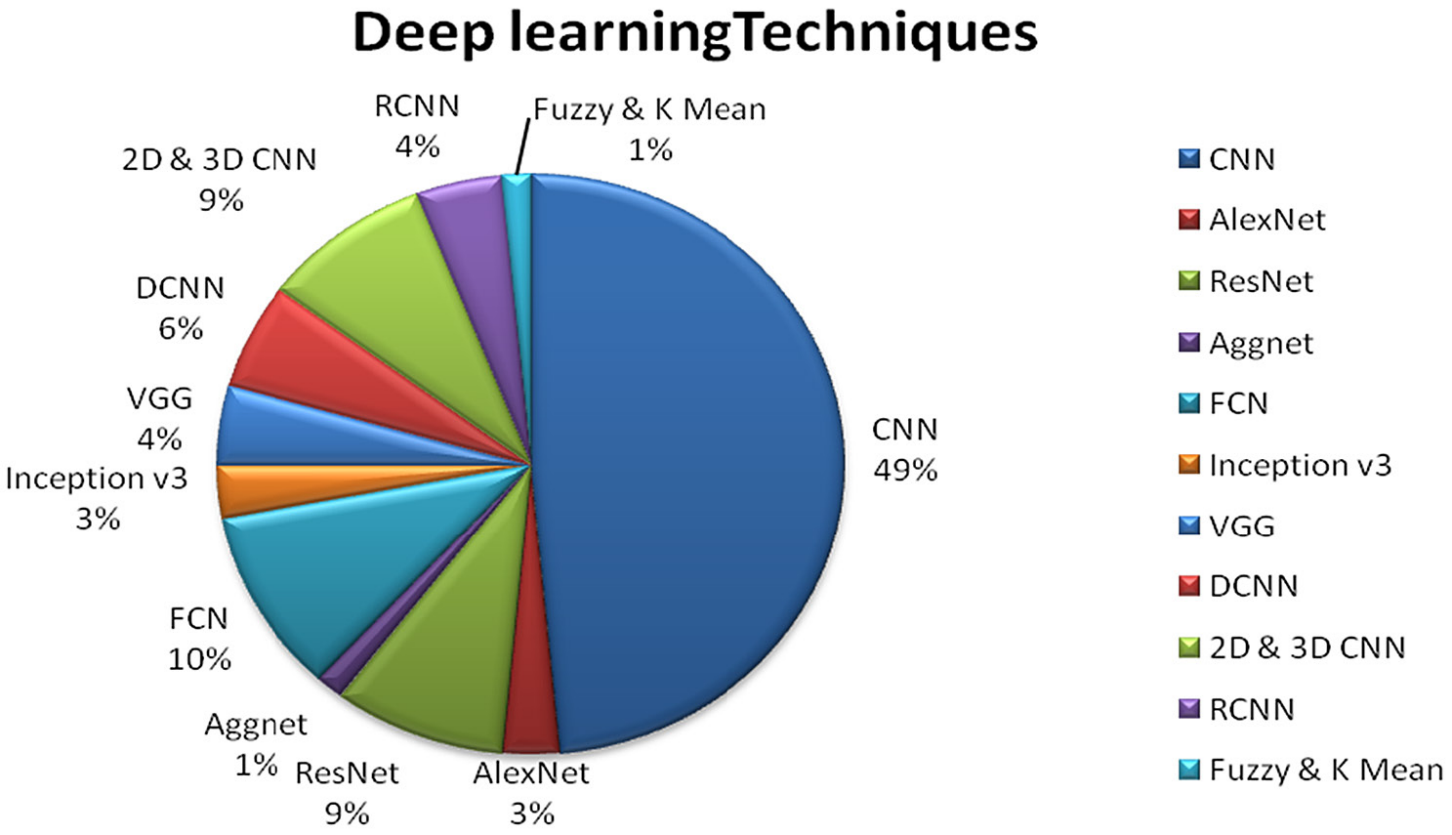
Distribution of publication on the basis of Deep learning techniques.

Deep learning‐based approaches have made major contributions to the field of cancer research. The research studies cited in the literature have primarily concentrated on deep learning approaches. In the realm of cancer research, deep learning classifiers have largely supplanted machine learning models. convolutional neural networks (CNN) has been the most often utilized deep learning model for cancer prediction; roughly 49% of researchers have used CNN to categorize cancer. Apart from deep learning approaches, the literature predominantly uses encoders, ensemble learning techniques (Random Forest Classifier weighted voting, Gradient Boosting Machines) and support vector machines (SVM). Figure 12 illustrates the distribution of articles using machine learning‐based prediction methods.

### 4.2 Cancer, type of data and imaging techniques used for detection

**FIGURE 13:**
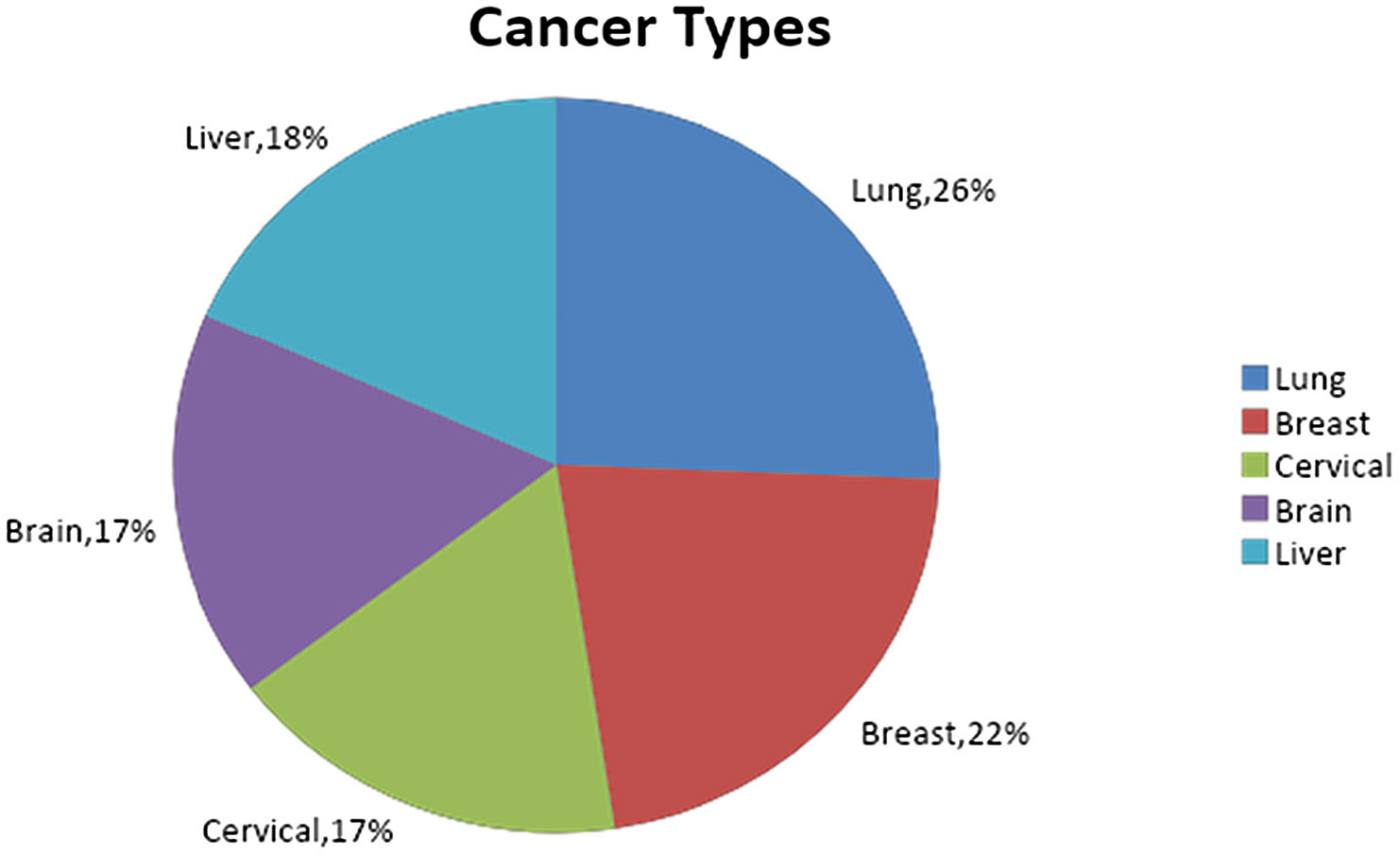
Distribution of publication based on cancer types.

The majority of the research publications included in this review concentrated on automated cancer detection and prediction. Lung cancer is the most widely used cancer, followed by breast cancer. Apart from lung and breast cancer, the majority of researchers have focused on the prediction of brain, liver and cervical cancer also. Figure 13 illustrates the distribution of research publications by cancer type.

**FIGURE 14:**
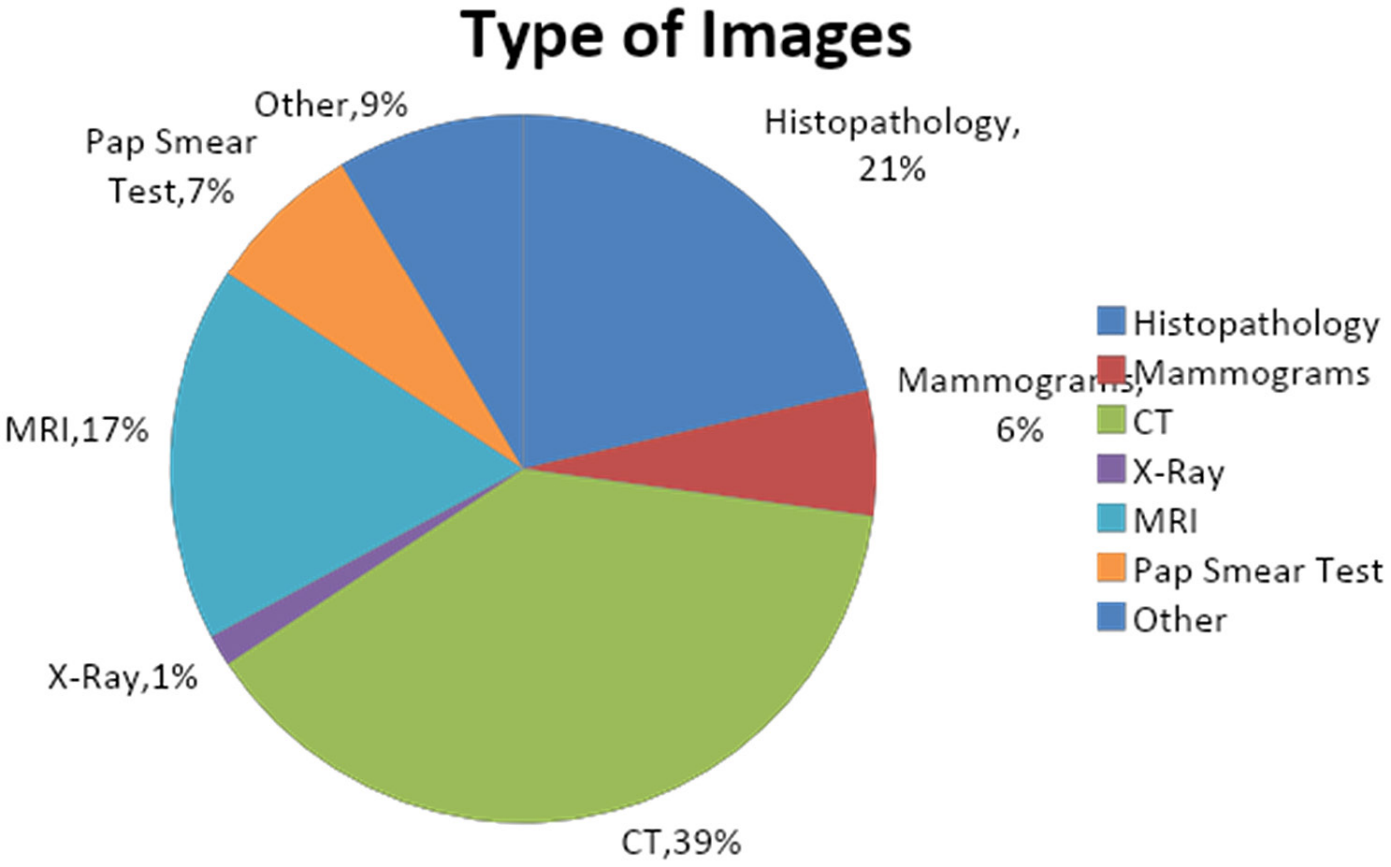
Distribution of publication based on the type of images.

The type of data utilized to train the prediction model has a major impact on the model's performance. The reliability and accuracy of the classification model are highly dependent on the data used to train it. Additionally, mammographic, endoscopic and pathological images were employed in the literature. The distribution of publications according to the type of data used to train the prediction model is highlighted in Figure 14.

### 4.3 AI‐based methods for consistent prediction outcomes

**FIGURE 15:**
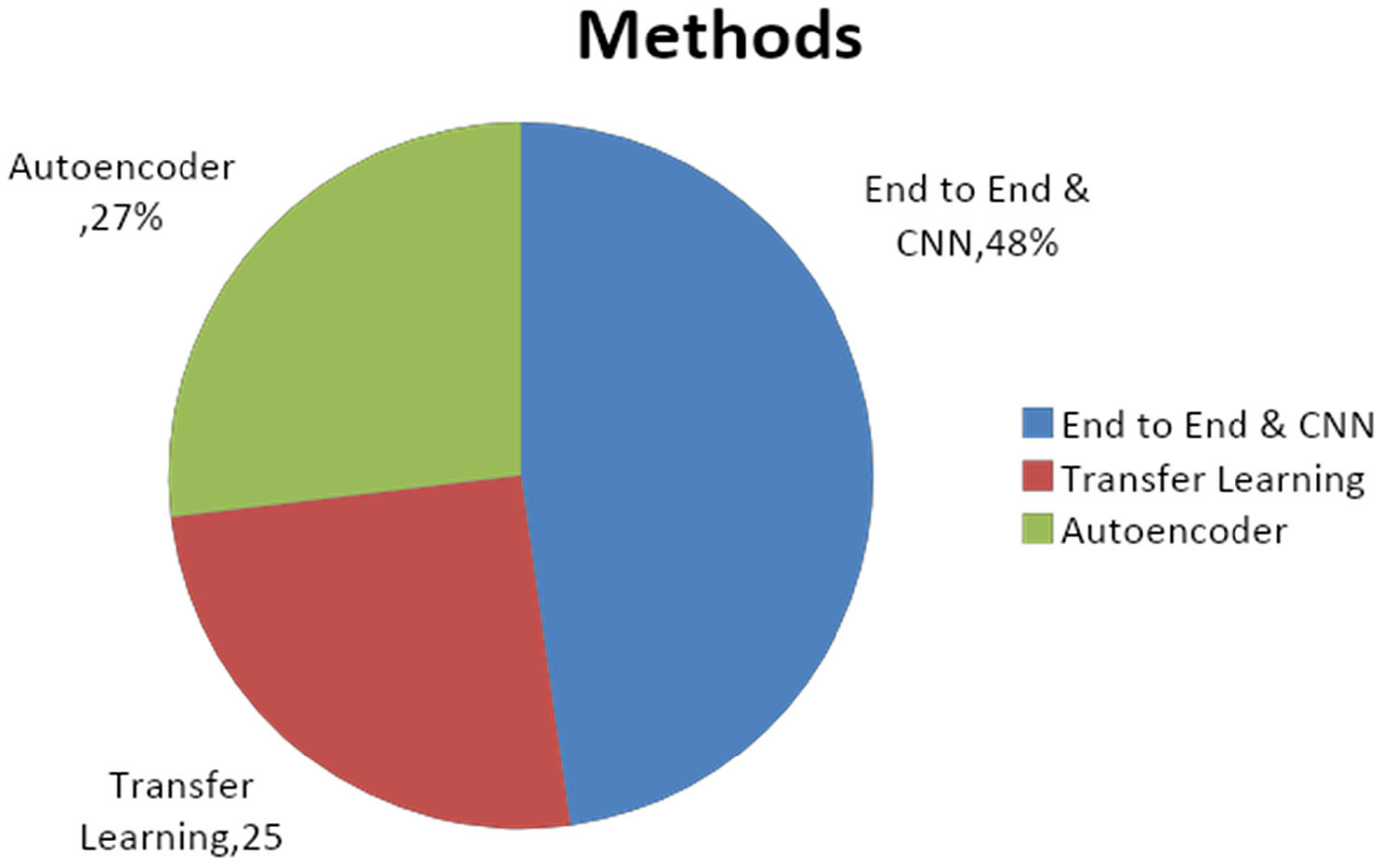
Distribution of publication on the basis deep learning methods.

Various deep learning methods like end‐to‐end learning and CNN, transfer learning, auto‐encoders and ensemble learning are used by the researchers for cancer detection. After the study, we found that among all included papers of this survey most of the authors used a transfer learning approach. The distribution of publications according to the AI‐based methods used to train the prediction model is highlighted in Figure 15.

### 4.4 Objective for using deep learning in cancer prediction

**FIGURE 16:**
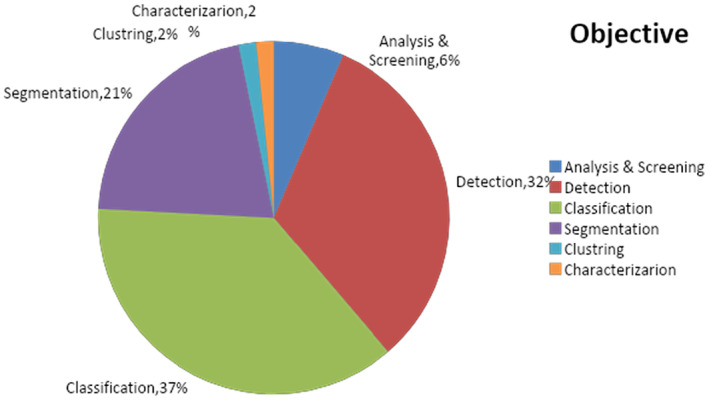
Distribution of publication on the basis of objective.

Different objectives like detection, classification and segmentation are taken by the researchers to predict the cancer. In this study, most of the researchers have worked on the detection objective. The distribution of publications according to the objective of study used is highlighted in Figure 16.

### 4.5 Data set and mode (online and offline) used in prediction of cancer

**FIGURE 17:**
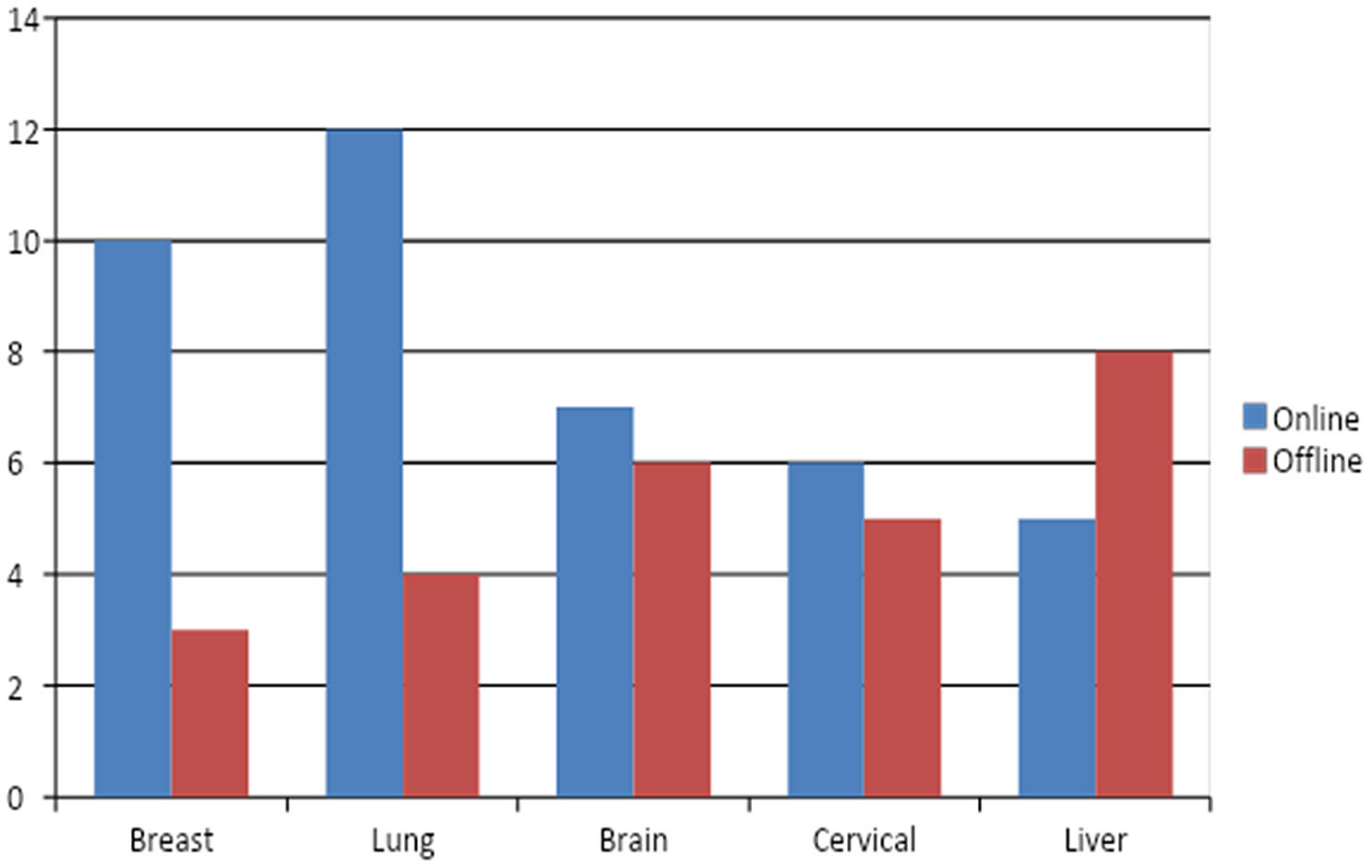
Distribution of publication on the type of data set.

The authors have used various types of data sets that are available both online and offline. In breast cancer most of the authors have used Break His data set, LIDC_IDRI data set in lung cancer, BraTs data set in brain cancer, and in liver and cervical cancer most of the authors have used offline data set or the data set collected from the private hospitals that are not available publicly. The distribution of publications according to the data set and mode is highlighted in Figure 17. The numbers of commonly available open source data sets for review are used in this study. Table 6 shows the summary of available open source data sets.

**TABLE 6:** Summary of data sets.

Data set	Description	Dataset source
MITOS ATYPIA‐14	This data set contains the breast cancer biopsy slides scanned by two slide scanners: A perio Scan scope XT and Hamamatsu Nano zoomer 2.0‐HT.	https://mitos‐atypia‐14.grand‐challenge.org/Dataset/
Break‐His	This data set contains a breast cancer histopathological image containing 9109 microscopic images of breast using different magnifying factors (40×, 100×, 200× and 400×).	https://web.inf.ufpr.br/vri/databases/breast‐cancer‐histopathological‐database‐breakhis/
MICCAI‐AMIDA‐13	This data set contains 500 breast cancer cases from The Cancer Genome Atlas.	http://tupac.tue‐image.nl/node/3
SEER	This data set is available in the binary format required by the SEER*Stat software and in an ASCII text format.	https://seer.cancer.gov/data/access.html
NLST	The National Lung Screening Trial (NLST) is a randomized controlled experiment containing images of chest X‐ray and CT scans.	https://cdas.cancer.gov/datasets/nlst/
LIDC‐IDRI	This Lung Image Database Consortium image collection (LIDC‐IDRI) contains 244,527 Dicom CT images of Lung.	https://wiki.cancerimagingarchive.net/display/Public/LIDC‐IDRI#194132fe653e4a7db00715f6f775c012
JSRT	The Japanese Society of Radiological Technology (JSRT) contains 154 nodule and 93 non‐nodule images	http://db.jsrt.or.jp/eng.php
BraTS	This Brain tumour segmentation (BraTS) data set contains multimodal MRI scans that are available as NIfTI files (.nii.gz).	https://www.smir.ch/BRATS/Start2013
Intel and Mobile ODT	This cervical cancer screening data set contains images of cervix for classification of cervical cancer to identify cancerous or noncancerous.	https://www.kaggle.com/c/intel‐mobileodt‐cervical‐cancer‐screening/data
BUPA Liver Disorders	This BUPA liver disorder data set contains blood test results and liver disorder status of 345 individual male patients.	https://archive.ics.uci.edu/ml/datasets/Liver+Disorders
LiTS	This liver tumour segmentation challenge data set contains a training data set of 130 CT scans and testing data set of 70 CT scans.	http://academictorrents.com/details/27772adef6f563a1ecc0ae19a528b956e6c803ce

The limitations of this study are as follows:

1. Publication bias: Publication bias could have an impact on the evaluation since papers showing promising outcomes are more likely to be published, which could result in an overestimation of the usefulness of deep learning models in cancer detection.
2. Heterogeneity of studies: FIt can be difficult to directly compare the performance of different deep learning models due to variations in the data sets, techniques and evaluation measures across different research. This might result in potential discrepancies and make it difficult to draw firm conclusions.
3. Ethical considerations: The ethical implications of depending exclusively on machine‐driven diagnoses, patient privacy, data security and other issues are brought up by automated cancer diagnosis models. To protect patient confidentiality and autonomy, these ethical issues must be carefully considered.

## 5 FUTURE SCOPE AND CHALLENGES

There is still potential for evolution to be done with using DL models to find and diagnose cancer cells. After the survey of various papers related to cancer detection using DL, it is observed that the major challenge to train the DL model is the lack of data. Most of the papers include confidential information of patients that they had collected from the hospital and that data are not publicly available. In order to make those private data available for research purposes, data de‐identification and data transportation are required. Many of the researchers used the data of the patients from the different hospitals or the cancer institutions. So it is very difficult to compare the performance of the DL models using that data. So, to achieve better accuracy data samples should be adequate to train, validate and test a DL model.

Some other future challenges in using the DL for medical image analysis are as follows:

*Preparation of High Resolution Data set*: For the training of DL model high resolution data set is required to perform better prediction. Although, the availability of that type of systems to confine and prepare such a large data set is still a challenge for researchers.

*Storage of large amounts of high resolution medical images*: Even though continuous improvement in terms of processing power and memory capacity, availability of compatible hardware resources to store the high resolution medical images is still a challenge.

*Selection of suitable model*: Proper selection of suitable model for capturing needed information from medical images and in terms of better accuracy.

*Selection of Suitable tools and technologies*: As medical images contain hidden information and cancer cells are having very small size so proper tools are necessary to extract hidden information from medical images.

*Deep transfer learning*: The use of deep transfer learning models is neglected in the literature. Therefore, to enhance the results further and to prevent underfitting kind of issues, in near future new models can be designed by using the deep transfer learning and deep belief networks.

*Hyper‐parameters tuning*: Additionally, the hyper‐parameters tuning issue is found to be a challenging issue in the literature, therefore, in near future meta‐heuristic techniques such as particle swarm optimization,^99^ can be used to tune the initial parameters of the models automatically.

*Image filtering*: Filtering models can be used to enhance the visibility of used images for cancer detection and diagnosis processes. Some of these filters are as trilateral filter,^97^ data cleaning, gradient filter, quantile separated histogram equalization etc.

*Multiobjective fitness function*: To tune the hyper‐parameters of the existing cancer detection and diagnosis approaches various multiobjective optimization techniques can be used such as Non‐dominated sorting genetic algorithm‐III, parallel strength Pareto evolutionary algorithm‐II, multiobjective genetic algorithm, memetic differential evolution, etc.

## 6 CONCLUSION

A systematic review of various automated computational deep learning‐based cancer diagnosis models was presented. Five types of cancers such as breast, lung, cervical, brain and liver at an early stage were studied. In total, 686 articles were selected that were published between 2016 and 2022 from five major databases such as Elsevier, Wiley, IEEE, Springer Nature and Nature portfolio. Out of these papers, by using systematic selection criteria, a selection of 113 articles is done for further reviews. Comparisons were drawn among competitive automated computational cancer diagnosis models. Various tools that have been used to build automated computational models were also discussed. Various shortcomings of the competitive automated computational cancer diagnosis models were presented. Finally, a future research direction in the field of automated cancer diagnosis models is discussed.

## AUTHOR CONTRIBUTIONS

**Ritu Tandon:** Conceptualization (equal); formal analysis (equal); investigation (equal); methodology (equal); resources (equal); writing – original draft (equal); writing – review and editing (equal). **Shweta Agrawal:** Formal analysis (equal); investigation (equal); project administration (equal); supervision (equal); validation (equal). **Narendra Pal Singh Rathore:** Conceptualization (equal); data curation (equal); formal analysis (equal); software (equal); writing – original draft (equal). **Abhinava Mishra:** Conceptualization (equal); investigation (equal); project administration (equal); resources (equal); supervision (equal); writing – review and editing (equal). **SANJIV KUMAR JAIN:** Project administration (equal); supervision (equal); visualization (equal); writing – original draft (equal); writing – review and editing (equal).

## FUNDING INFORMATION

‘This research received no external funding’.

## CONFLICT OF INTEREST STATEMENT

The authors confirm that there are no conflicts of interest.

## Data Availability

The data that support the findings of this study are available from the corresponding author upon reasonable request.
